# Supplementary material for: Are carbon emissions trading and green financial instruments synergistic? -Comprehensive quantitative research based on content analysis
Source: PLoS One. 2024 Mar 7;19(3):e0298601. doi: 10.1371/journal.pone.0298601 (PMC10919601; doi:10.1371/journal.pone.0298601)
Supplement: S1 Data — (ZIP) [file pone.0298601.s001.zip › Data/地级市Z_F工作报告.zip!/╜¡╬≈/╔╧╚─╩╨/└┤╫╘╣1⁄2╓┌║┼í╛┬φ┐╦╩2╛▌═°í┐_2005.docx]

各位代表：

现在，我代表市人民政府向大会作工作报告，请予审议，并请各位市政协委员和列席会议的同志提出意见。

一、2004年工作回顾

过去的一年，全市人民在中共上饶市委的领导下，以科学发展观为指导，认真贯彻国家宏观调控政策，紧紧围绕“建设赣浙闽皖四省交界区域中心城市和快速发展地区”的战略定位，大力实施“掉头向东，通江达海，对接长珠闽，实现大发展”的发展思路，开拓进取，扎实工作，经济社会继续保持快速、健康、有序的发展势头，全面和超额完成市一届人大七次会议确定的各项任务，主要经济指标提前一年完成“十五”计划。

——经济发展速度进一步加快。全市生产总值突破300亿元，达到301.1亿元，增长14.5%，为1996年以来最大增幅。财政总收入26.8亿元，增长24.5%，其中一般预算收入17亿元，增长24%，增幅居全省第三位。

——投资、消费、出口的拉动力进一步增强。全社会固定资产投资152.8亿元，增长35.7%；社会消费品零售总额123.8亿元，增长16.1%；外贸出口总额1.67亿美元，增长107%，实现了“两年翻两番”。

——经济结构进一步优化。三次产业结构由上年24.8∶36.7∶38.5调整为21.5∶41.0∶37.5，二产比重首次超过三产。工业占GDP比重达31.9%，同比增加2个百分点；经济外向度、城市化率分别达5.02%、25%，分别提高2.01和2.3个百分点。

——城乡居民生活进一步改善。城镇居民人均可支配收入7573元，增长11.8%，农民人均纯收入2912元，净增529元，年增加额创历史最高水平。

——社会事业发展进一步协调。全市有23万人参加养老保险，25万人参加失业保险，24万人参加职工医疗保险，12万人享受城市居民最低生活保障，17万农村特困群众列入救助对象，从库区、偏僻山区移民6001人，城镇新增就业5万人，城镇登记失业率3.35%。人民群众对社会治安满意度达到95.8%，外商对上饶投资环境满意度达到90.5%。

一年来，我们主要做了以下工作：

第一，始终把“三农”工作作为重中之重。坚定不移地贯彻中央、省委两个“一号文件”精神，全面落实“两减免三补贴”政策，出台促进粮食增产和农民增收的政策措施29条。继续加大农业结构调整力度，发展高产、优质、高效农业，推进农业产业化经营，农业和农村经济发展呈现多年没有的好形势，实现粮食播种面积、粮食产量和农民收入分别增长21.4%、27.1%和22.2%“三个20%以上增长”，粮食总产51.7亿斤，创历史新高；农民收入增幅首次超过城镇居民收入增幅。养殖业产值占农业总产值比重达到45.2%，同比提高1.6个百分点；各类农业企业新增264家，总数达到2000家，德兴异VC钠有限公司晋升为国家级龙头企业，国家、省、市级龙头企业达到48家；绿色食品新增50个，总数达到95个，占全省近三分之一。

第二，在加强宏观调控中推进重点项目建设。坚持适时适度、区别对待、有保有压、注重实效的原则，开展治理整顿土地市场秩序和清理固定资产投资项目工作，全市15个工业园区整合为11个，规划面积由51.7万亩调减到6.4万亩。与此同时，在宏观调控中抢抓机遇，争取国家投资，推进基础设施重点项目建设。交通建设，景婺黄（常）高速公路已开工建设，宁德至上饶高速公路已经国家立项建设，昌万、白景、上广公路等建成通车，鄱余公路、三清山旅游公路等正在加紧建设，全年改建快速通道184公里，改造农村公路1240公里，较上年增加590公里。电力建设，黄金埠电厂顺利开工，玉山、弋阳、余干等县22万伏输变电站基本建成，新建11万伏输变电站4座，新建、改造农网35千伏变电站66座，在建水电工程66座，新增水电装机容量8.6万千瓦时。水利建设，鄱阳湖防洪二期治理四个单项工程基本建成，饶河大堤、康山大堤等8条重点圩堤通过省级验收，枫富联圩除险加固、信州水利枢纽、高店水利枢纽以及上饶、弋阳、婺源等县城市防洪工程正在实施之中，军民、滨田、蜈蚣山等大中型水库以及157座小（二）型水库得到除险加固，平垸行洪、退田还湖、移民建镇工作基本结束。通讯设施建设，组建信息网络办公室，开通政务信息网，实现省、市、县政务三级联网；新增固定电话用户16.9万户、移动电话用户16.1万户、宽带互联网用户2.7万户。生态建设，完成退耕还林、长防林75.6万亩，实施国家重点生态公益林257.7万亩，完成水土保持综合治理38万亩。加强环境保护，全市环境质量得到改善，地表水优于 Ⅲ类水比例上升9.3%；城市环境空气综合污染指数降低0.61。

第三，坚定不移地实施工业化核心战略。全市工业实现“三年翻一番”目标，规模以上工业增加值38亿元，增长35.3%；销售收入113.5亿元，增长48.2%；利税总额10.3亿元，增长43%。来自工业的财政收入14.8亿元，占全市财政总收入比重达到54.3%，工业用电量24.9亿度，增长19%，其中地方工业用电量10.1亿度，增长52%。进一步加大工业园区建设力度，整合园区资源，实现旭日、三江、凤凰“三园合一”，注重园区招商效果，强化管理与服务，提高园区集约化水平。全市工业园区新增入园企业368个，新增投产企业157个，工业增加值、销售收入、实交税金分别增长155.5%、147%和75%。进一步加大民营工业发展力度，出台一系列政策措施，营造全社会踊跃创业氛围，规模以上民营工业企业销售收入增长92.2%，占全市地方规模以上工业总量51.6%；纳税过百万元民营企业达到90家，新增16家，其中过500万元民营企业达到14家，新增10家。进一步加大重点企业和支柱产业培育力度，列入重点调度的35户重点工业企业销售收入增长20.6%，年销售收入过亿元企业达到19家，新增7家；机械、建材、食品、有色金属、纺织服装和医药化工等六大支柱产业增加值、销售收入、利税总额分别增长46%、61.6%和47.3%，分别占全市规模以上工业总量的71.6%、69.8%和74.9%。

第四，进一步加快“通江达海、对接长珠闽”步伐。积极主动参与和融入长珠闽经济技术合作，成功加入长三角“15+1”旅游经济圈，密切与泛珠三角“9+2”城市联系。进一步加大招商引资力度，先后组团到美国、欧洲、东南亚、日本和北京、长三角、珠三角等国内外主要城市召开经贸恳谈会，实现开放型经济新突破。全年实际引进市外资金157.5亿元，增长36.4%；实际利用外资1.32亿美元，增长65.5%，增幅列全省第一位。进出口贸易总额达1.82亿美元，成为全省3个利用外资和出口总额双亿元设区市之一。全市共接待国内游客450万人次，增长38.9%，国内旅游收入23.12亿元，增长21.9%；接待入境游客1.2万人次，增长35.5%，旅游外汇收入452万美元，增长15.9%。

第五，始终坚持把改革创新作为经济发展强动力。推进国有资产管理体制改革，组建国有资产管理委员会；撤并市机电、轻化纺、建材、煤冶等四家国有资产经营公司，组建市工业国有资产经营公司和市煤炭行业办公室。整合红色旅游资源，组建上饶集中营名胜区管委会和方志敏纪念馆·闽浙皖赣革命根据地旧址管委会。整合文化教育资源，设立上饶职业技术学院，上饶学院的组建工作正在有序进行。整合市区公共卫生资源，组建市疾病预防控制中心和市卫生监督所。推进公路建设和交通管理体制改革，在全国率先组建汽车经济服务中心，在全省率先编制城乡公共交通规划，构建中心城市城乡公交一体化。深化农村金融改革，成立全省首家农村合作银行，全市金融形势良好，新增贷款首次超过新增存款，新增贷款总量和增幅均居全省第二位。深化投融资体制改革，形成多元化投融资机制，全社会固定资产投资中来自非国有单位投资占56.83%，提高11.4%。财税管理体制改革进一步推进，农村新型合作医疗和林权改革试点取得成效。

第六，努力促进区域协调发展。出台加快滨湖地区发展和推进城镇化进程的政策措施，区域发展进一步协调。12个县（市、区）财政收入超亿元11个，其中超5亿元、3亿元各1个，超2亿元2个；财政收入超过500万元乡镇由上年31个增加到40个，其中1000万元以上乡镇由14个增加到24个。坚持规划先行、科学管理，不断加快中心城市建设。市、县两级投资6000多万元编制各项规划，在全省率先编制市域城镇体系规划，完成市工业园区、旅游发展、行政新区核心区、大学新校区等20项重点项目规划，市区建成区面积拓展8平方公里，达到35.2平方公里。着力实施一批城建重点工程，三清山大道、凤凰大道、滨江路、紫阳大道、五三大道东延伸段等道路改造顺利完工，中山路和滨江路“五位一体”、121条里弄小巷、信江南岸和水南街改造等工程已竣工使用，市区铁路北移、行政新区建设、污水处理厂等工程正在加紧实施。全市完成城建投资45亿元，增长12.5%，其中市本级22亿元，增长5%。

第七，推动社会各项事业共同进步。科教兴市战略扎实推进，实施一批重点科技攻关和科技推广项目，德兴市被评为全国科技进步示范市。农村义务教育得到加强，横峰县“普九”教育已达标验收，优质高中建设力度加大，民办和职业教育快速发展。卫生事业取得进展，市非典定点医院竣工使用，市医院综合住院大楼基本完工，12个疾病预防控制中心开工建设。文化事业繁荣发展，市图书馆已开工建设，赣剧《詹天佑》荣获国家文华新剧目奖，弋阳腔被列入国家民族民间文化保护工程试点项目。广电事业建设荣获全省八连冠，有线电视网络用户达48.7万户。广泛开展群众性体育活动，在全省第二届全民健身运动会上取得团体总分第三名。精神文明创建活动蓬勃开展，在城市开展卫生、园林、文明、双拥模范和优秀旅游城市“五城同创”活动，在农村开展创建“一个规划、一个环境、一个场所、一个体系、一个风俗”的“文明村镇五个一”活动，涌现出“网络妈妈”刘焕荣、文明城镇玉山仙岩镇、全国民主法治示范村婺源江湾村和全国百城万店无假货示范街抗建路步行街等一批先进典型。人口和计划生育工作深入推进，重点治理出生人口性别比失调问题，人口自然增长率控制在7.95‰以内。安全生产总体形势稳定，安全事故发生数和死亡人数下降8.2和7.9个百分点。“平安上饶”建设全面启动，深入开展以矛盾纠纷排查调处和治安专项整治为主要内容的“百日行动”，全市社会政治保持稳定。认真做好群众来信来访和人民调解工作，着力解决群众反映的突出问题。新闻出版、外事侨务、民族宗教、对台工作、地方志、档案、保密、气象、水文、防震减灾、民兵预备役和“双拥”工作、国防教育、人民防空、社会福利、妇女儿童、老龄、残疾人等事业健康发展。

第八，不断加强政府自身建设。围绕建设服务型政府，加强政府效能建设，修订《市政府工作规则》，完善考核机制，开展现场办公，强化督促检查，狠抓工作落实；密切与群众联系，市长热线共受理群众来电1.8万个，网上来函1462件，办结率均在93%以上。围绕建设法治政府，加强民主法制建设，完善行政监督机制，自觉接受人大及其常委会的法律监督、工作监督和人民政协的民主监督，认真办理人大代表议案、建议和政协委员提案，办结率100%。注重发挥各民主党派、工商联、无党派人士和人民团体的参政议政作用，广泛听取各方面意见，改进政府工作。贯彻实施《行政许可法》，共清理行政审批事项598项，保留行政许可事项291项。围绕建设廉洁政府，加强廉政建设，认真落实党风廉政责任制，深入开展反腐败斗争，加强预算外资金管理，规范政府采购行为，强化审计监督，推行政务公开、厂务公开、村务公开，开展评议市直机关活动，政风建设取得明显成效。

各位代表，过去的一年，我们朝着建设四省交界区域中心城市和快速发展地区的目标迈出了坚实步伐。这是省委、省政府和市委正确领导的结果，是市人大、市政协监督支持的结果，是广大老干部、老同志和驻饶各单位大力支持的结果，凝聚了全市人民的智慧、心血和汗水，凝结了社会各界、海内外朋友及在外工作乡友们的关心、支持和帮助，包含着在饶投资创业的客商朋友的贡献。在此，我谨代表市人民政府，向全市工人、农民、知识分子，向各界劳动者，向驻饶人民解放军、武警官兵、公安干警表示崇高的敬意！向所有关心支持上饶发展的同志们、朋友们和社会各界人士表示衷心的感谢！

回顾过去一年的工作，我们也清醒地看到，前进道路上还有不少困难和问题，当前经济社会生活中还存在一些突出矛盾。主要是：经济结构性矛盾仍然突出，经济增长方式需要进一步转变；农业综合生产能力仍然薄弱，农民持续增收的长效机制需要进一步培育；就业和再就业的压力仍然很大，社会保障工作需要进一步强化；影响社会稳定的因素仍然较多，构建和谐社会的力度需要进一步加大。从政府自身看，依法行政能力有待进一步提高，政风建设有待进一步加强。对这些问题，我们将高度重视，并在改革发展稳定中采取有力措施，逐步加以解决。

二、2005年工作任务

今年，是全面实现“十五”计划、衔接“十一五”发展的关键一年，也是我市加快建设四省交界区域中心城市和快速发展地区、全面建设小康社会的重要一年。根据市委一届十三次全会精神，政府工作要以科学发展观为统领，以保持共产党员先进性教育为契机，以建设四省交界区域中心城市和快速发展地区为目标，深入贯彻党的十六届三中、四中全会和省委十一届八次全会精神，以主攻工业为核心，坚定不移地实施大开放战略，坚定不移地推进工业化、城镇化和农业产业化，加快速度，做大总量，优化结构，提升质量，增强区域经济竞争力，促进“三个文明”协调发展，努力构建和谐社会，在新的起点上实现上饶经济社会更快更好地发展。

经济社会发展的预期目标是：全市生产总值比上年增长14%；财政总收入增长16%；全社会固定资产投资增长20%；社会消费品零售总额增长14%；实际引进市外资金180亿元，实际利用外资1.5亿美元，外贸出口2.25亿美元；城镇登记失业率控制在3.5%以内；城镇居民人均可支配收入增长10%，农民人均纯收入净增200元、增长7%以上；人口自然增长率控制在8.05‰以内，城市化率达到28%。

上述预期目标，是在全面分析宏观形势、综合考虑各种因素的基础上，本着既积极可行又留有余地的原则提出的。综观国内外形势，我们正处于必须紧紧抓住并且可以大有作为的重要战略机遇期，全球经济和贸易持续增长，外部环境总体上比较有利。国内经济仍处于新一轮增长周期的上升阶段，宏观环境进一步改善。我市经济已站到一个新的起跑线上，正在进入迈大步、登台阶、快发展的新阶段，自主增长活力不断增强，支撑加速发展的基础更加巩固。特别是省委、省政府把上饶作为全省四个重点发展城市之一，提出要把上饶建成江西东北部区域经济中心城市和对接长三角的前沿阵地，这是省委、省政府对上饶工作的充分肯定，是对上饶发展的殷切期望，也为上饶发展指明了方向。不发展不行，不加快发展不行，不科学发展更不行。在这关键的发展时期，只要把握得当，抢抓机遇，振奋精神，真抓实干，就一定能在科学发展道路上迈出新的步伐，创造新的业绩。

实现上述目标，要着重做好以下工作：

（一）加强“三农”工作，提高农业综合生产能力，保持农业和农村经济发展的好势头

稳定、完善和强化各项支农政策。坚决落实省委、省政府作出的从2005年起免征农业税的决定，继续对稻谷实行最低收购价政策，继续实施粮食直补、良种补贴和农机具购置补贴，争取中央和省对粮食主产区和财政困难县的支持。

加强农业综合生产能力建设。实行最严格的耕地保护制度，严格控制非农建设占用耕地，确保基本农田总量不减少、质量不下降、用途不改变，并落实到地块和农户。加大农田水利建设和中低产田改造力度，抓好病险水库除险加固、生态重点工程和农村基础设施建设，实施好土地整理、农业综合开发和农业现代化建设项目，打牢粮食增产和农民增收的基础。

按照“希望在山，潜力在水，重点在田，后劲在畜，出路在工”的要求，推进农业结构调整和农业产业化经营。进一步稳定粮食生产，实施好优质商品粮基地项目，提高粮食综合生产能力，力争粮食播面稳定在755万亩、总产稳定在51亿斤左右。以市场为导向，搞好农业产业规划，进一步优化特色优势农产品布局，大力发展项目农业、特色农业、高效农业和品牌农业，创建特色乡镇。用发展工业的理念发展农业，推动农业规模化、专业化、集约化和标准化生产。加快建设一批优质农产品基地，发展一批特色农业产业，扶持一批农业产业化龙头企业，培育一批优势农产品品牌，努力提高经济作物在种植业中的比重，提高畜牧业在农业总产值中的比重。加强农产品质量标准体系建设，大力发展无公害农产品和绿色有机食品。拓宽鲜活农产品“绿色通道”，提高农产品市场占有率。积极发展农产品出口基地和加工企业，扩大农产品出口。

加快农业科技创新和技术推广。进一步完善农业科技推广网络，建立健全农业植物保护、农业技术推广、农产品检验检疫和动植物疫病防治体系，抓好农民素质培训，提高农村劳动力转移和就业能力；建设农民工跨地区就业服务体系，促进农村富余劳动力转移。

（二）主攻工业，加快产业集聚，加速工业化进程

深入贯彻《关于实施主攻工业战略的若干意见》，抓住园区、产业、企业三要素，进一步加大主攻工业力度，加快产业建设，坚持以信息化带动工业化，走新型工业化之路。

集中力量抓好工业园区建设。按照土地集约经营、产业链延伸、企业集群组合、资源循环利用的要求，提升园区开发水平，增强园区产业集聚、就业吸纳、环境承载、要素集约和自主创新能力。重点打造上饶工业园、芦林工业园，力争这两个工业园在2007年前后年销售收入分别达到100亿元以上；加快建设凤凰光学产业园、玉山建材和轴承工业园、博能工业园、奥得电子电器产业园等特色园区以及省级大茅山经济开发区。着力抓好入园企业达产达标，对进资额过2000万元工业项目实行重点调度，力争园区年销售收入、上交税金翻一番。

集中力量培育机械、建材、食品、有色金属、纺织服装、医药化工等六大支柱产业。加快实施三和金业生物氧化提金、金山金矿扩建、花桥金矿扩建、德兴新华公路改性沥青、鑫新实业特种漆包线、万年青万年5#窑新建、广丰永利熔体直纺和福丰氟化工等一批重大工业项目，加速推动玉山鸡山和岩鹰日产2500吨水泥生产线、广丰台鑫45万吨钢材生产线、弋阳江南涤化、铅山年产40万吨和德兴年产20万吨硫酸以及杭州味精厂整体搬迁至余干等项目建设，抓紧建设精元电脑、成功信息产业、泽众制药等一批高新技术项目。着力培育一批重点骨干企业，力争年销售收入过亿元企业达到30户。大力发展农产品加工业，力争每年新增规模以上农产品加工企业10家以上。大力实施质量兴市和品牌战略，增强上饶工业产品竞争力。

集中力量推进企业技术进步。围绕提高自主创新能力，加快技术创新步伐，加大新技术、新产品研发、引进及推广应用力度，加快用高新技术、先进适用技术改造提升传统产业。抓紧实施制造业信息化工程，着力实施4家省级和13家市级应用示范企业信息化项目，推动传统制造业信息化。

（三）做大做强商贸、旅游业，提升服务业发展水平，加大消费对经济增长的拉动力

坚持改造提升传统服务业和加快发展现代服务业并举，大力发展商贸流通业。围绕建设区域中心城市，搞好商贸网点规划和布局，发展各种专业市场，建设一批商品批发市场，加快上饶商品大市场、月亮湾汽车城、江南商贸城、国际家具城、江南米业市场、鄱阳湖国际水产城等重点市场建设和服务设施配套。依托立体交通网络，发展现代物流业，加快建设华欣物流园和新华龙货运服务中心，推进物流枢纽、园区和配送中心建设，建立专业化、社会化物流服务网络。进一步发展宾馆、餐饮、邮电通讯、交通运输、保安保绿保洁等传统服务业。顺应消费趋势，着力培育汽车、住房、文化娱乐、教育培训、体育健身、卫生保健、社区服务等消费热点，扩大服务性消费。深化商贸流通企业改革，大力发展连锁经营、商业超市等新型业态，加大商贸流通业招商力度，争取大型跨国零售企业集团到我市投资和经营。进一步整顿市场秩序，加强市场监管，实施“食品药品放心工程”，改善消费环境，引导消费预期，增加即期消费。鼓励超市下乡，搞活农村流通，开拓农村消费市场。

积极培育资本市场，发展金融、保险业。支持各金融、保险、证券机构在我市开展公平竞争的经营活动，支持金融机构进一步调整信贷结构，增加企业生产必需的流动资金贷款。加强社会信用体系建设，打造信用上饶，重视防范和化解地方金融风险。

致力把旅游业培育成市域经济的支柱产业。抓住加入长三角旅游经济圈的新机遇，在资源共享、互送客源、发展机制等方面全方位融入长三角，打造无障碍旅游区。坚持政府主导与市场运作、景区开发与城市建设相结合，加强对旅游产业规划，实现旅游资源可持续开发。强化旅游招商，进一步整合红色、古色、绿色旅游资源，建设上饶旅游精品。启动三清山申报世界自然文化遗产工作，加大创建文明景区力度，力争使三清山成为全国首批文明风景旅游区。围绕创建“中国优秀旅游城市”目标，在旅游规划、宣传促销、设施建设、市场规范和队伍建设等方面下功夫，优化旅游环境，开拓客源市场。

（四）推进重点项目建设，完善基础设施体系，进一步增强经济发展后劲

以项目为抓手，进一步完善基础设施体系。大力实施交通畅通工程和城乡公交一体化，年底基本建成中心城区到周边五县城的公交网络，实现市本级“半小时交通圈”内城乡公交一体化。加快建设景婺黄(常)高速公路，确保年内路基工程全面完成；开工建设景鹰高速公路；继续做好宁德至上饶高速公路前期工作；开工建设上乐公路和岑葛红色旅游公路；加快鄱余公路、乐江线等公路建设；建设快速通道90公里，改造农村公路950公里，争取开工建设余干龙津大桥、中洲大桥和鄱阳莲湖大桥等6座渡改桥工程，加快农村汽车客运站和城市公交候车亭建设；积极配合国家完成浙赣铁路电气化改造，完成火车站主体站房的基础建设，争取九景衢铁路立项建设。加快通讯设施建设，努力提高城乡通信网络覆盖率。进一步加强水利设施建设，加快信州水利枢纽建设，确保5月底下闸蓄水；加快市区、玉山、德兴、婺源、弋阳、万年等县城市防洪工程建设；抓好鄱阳沿河圩、余干枫富联圩和高店水利枢纽建设，开工建设伦潭水利枢纽工程，实施鄱阳湖区、信江尾闾区河湖疏浚、鄱阳湖区机电泵站更新改造和广丰关胜灌区节水灌溉示范项目，争取完成200座病险水库除险加固。继续加强电力建设，加快建设黄金埠电厂，启动50万伏输变电工程，全面改造县城电网，建设茅家岭、洪家嘴22万伏输变电、贵溪至弋阳22万伏输电线路和丁家洲、旭日、五府山、铅山紫溪11万伏输变电工程，推进在建的三个22万伏变电站的建设。积极鼓励发展农村小水电，新增装机9万千瓦。

（五）统筹城乡发展，加快区域中心城市建设，有序推进城市化进程

按照“五个统筹”要求，进一步贯彻市委关于加快滨湖地区发展和加快城市化进程的两个《决定》，以滨湖地区快速崛起支持上饶崛起，以工业化和城市化互动共进促进上饶加快发展，确保城市化率提高3个以上百分点。

加快中心城市建设，强化中心城市功能。在中心城区远景控制的140平方公里范围内打破体制局限，统一规划、统一布局、统一土地资产管理，实现土地资源效益最大化。加大规划力度，进行城市总规修编、土地利用总规修编等15项规划编制工作，强化规划执行的监督、管理。在继续推进续建项目基础上，重点推进十大城建工程，即：开工建设凤凰大桥、三清山大桥、行政中心大楼、三江污水处理系统工程和市垃圾处理厂，改造滨江西路及双塔景观、水南街东环路及与上广一级公路交接点景观、城市中心广场和122条里弄小巷，续建信江南岸景观改造工程。同时进一步加强城市配套和公用设施建设，搞好城市绿化、美化、亮化和净化，提升城市品位。强化城市管理，推行综合执法和城市管理中心下移，提高城市管理水平。

加快小城市群建设。按照“绿色空间分隔、快速通道相连、功能设施互补”的要求，建立联动发展机制，加强规划协调，合理产业布局，搞好城市产业分工，完善城市功能设施，加速形成以市区为中心的“上广玉信弋横铅”“半小时经济圈”、“鄱余万”滨湖城市群和“婺德”特色城市。

加快以县城为核心的小城镇建设。以拉开城市道路框架、完善城区道路交通网络为重点，加快县城基础设施建设，及时跟进市场、学校、宾馆、医院、体育、文化等社会配套项目，强化县城功能。加强对县城镇、重点集镇和中心村的规划、编制和管理，重点指导、支持财政收入过400万元、镇区人口8000人左右的重点镇、门户镇、边贸镇建设，深入开展文明村镇创建活动，塑造城镇新形象。抓好人口超3000人的中心村村庄规划、村容村貌和村经济发展，在全市率先建成全面小康示范村。

（六）深入实施大开放主战略，进一步加大招商引资力度，不断提高经济对外开放度

主动对接长珠闽，融入全球化。积极参与国内外特别是沿海地区经济技术合作，进一步加强与友好城市、周边城市的联系和交流。抓好侨情普查，发挥侨界优势，主动和国外城市缔结友好关系，更多地参与全球产业分工和经济合作。

进一步加大招商引资力度。深入开展重大项目招商引资年活动，切实落实工业、农业、基础设施、旅游和科教文卫等“五条线”招商引资任务，加强重点产业、区域、企业的招商，积极参与全省组织的对外招商活动，落实各项合作协议，建立健全重大招商项目合作协调机制，突出抓好投资规模2000万元以上项目的跟踪落实，努力提高招商引资质量和水平。

努力扩大外贸出口。加快培育多元化出口主体，扶持一批规模大、效益好的外贸企业集团。抓紧上饶海关申报和建设，加快构建与发达地区产业协作平台，培育有比较优势的配套协作项目，推动加工贸易延伸产品链，培植新的外贸出口增长点。

积极主动地“走出去”。扩大对外劳务输出和经济技术合作，支持博能集团等企业到国外直接投资，增强创汇和盈利能力。建立有效应对技术壁垒、反倾销等工作机制，积极应对加入世贸组织过渡期后国际资本、市场的变化，抢抓机遇，主动融入全球经济。

（七）稳步推进各项改革，加大资源整合和机制创新力度，增强经济发展活力

以产权制度改革为重点，深化国有企业改革，完善国有资产管理机制和监管方式。完善凤凰、旭日、三江工业园整合机制，确保市工业园区各项管理到位。毫不动摇地鼓励、支持和引导非公有制经济发展，创新机制，激活全社会创业细胞，让一切有利于群众创业的思想活跃起来，让一切有能力创业的人才脱颖出来，让一切领域的创业热情充分发挥出来，形成全社会踊跃创业的浓厚氛围。继续深化农村税费改革，加快推进乡镇机构改革、农村义务教育体制改革，精简乡镇机构，减少财政供给人员。积极稳妥地推进事业单位人事制度改革。进一步深化粮食购销市场化改革，全面启动林业产权制度、水利工程管理体制改革。依法稳定农村土地承包关系，搞好征地制度改革。积极推进市交通体制改革，实施市本级城乡公交一体化，整合交通产业资源，组建交通投资（集团）公司。深化部门预算、国库集中收付、政府采购和“收支两条线”管理改革，逐步建立业务监督和专业监督相结合的财政监督运转机制。推进投融资体制改革，全面落实国务院关于深化投资体制改革的决定及其相关配套政策。进一步改进和规范投资审批和核准方式，落实企业投资自主权。

（八）坚持以人为本，促进经济社会协调发展，努力构建和谐社会

大力培育社会主义先进文化。推进精神文明建设，弘扬方志敏精神，加强思想道德建设，强化社会公德、职业道德、家庭美德教育，形成积极向上、健康和谐的社会氛围。深入推进科教兴市战略，进一步强化科技是第一生产力观念，以科学人才观为指导，以能力建设为核心，加快各类紧缺人才特别是技能型人才培养。加强创新体系建设，重点扶持一批高新技术项目，鼓励、支持民营科技企业发展；进一步探索科技兴农、科农结合的途径，扩大科技特派员试点工作。加快教育资源整合步伐，在上饶师院基础上组建上饶学院，加快万年师范的转型；以上饶职业技术学院为龙头，大力发展职业和成人教育，鼓励发展民办教育；加大农村教育扶持力度，确保余干、鄱阳两县年内通过“两基”评估验收。加快市图书馆建设，完善农村文化基础设施，丰富群众文化生活；组团参加省第三届艺术节，举办四省四市民族民间文化艺术节。深入开展全民健身运动，办好首届全市运动会。加强公共卫生建设，全面完善疾病预防控制体系、救治体系和社区、农村卫生基础设施，重点加强血吸虫病、结核病等重大疾病防治工作，加强食品、药品安全监督，切实保障群众的身体健康和生命安全。进一步抓好人口与计划生育工作，稳定计划生育政策和低生育水平，提高人口素质，改善人口结构，坚决遏制性别比失调的问题。抓紧新体育场等基础设施建设，广泛开展全民健身运动，不断增强人民体质。进一步做好老龄和残疾人工作。继续深入开展“五城同创”活动，力争今年市区创建成省级卫生、园林、文明和“双拥”模范城市。进一步加强全民国防教育，依法开展兵役工作和民兵教育训练，促进经济和国防后备力量建设协调发展。

切实办好民生大事。进一步加强社会保障工作，加快社会保障体系建设，推进国有企业下岗职工基本生活保障向失业保险并轨，依法扩大养老、失业、医疗、工伤等社会保险覆盖面，提高个体、私营和外资企业的参保率。注重解决城乡困难群众基本生活问题，建立以城市低保、农村五保、特困户救助制度为基础，临时社会救助为补充的社会救助体系，做好优待抚恤工作，支持社会福利事业发展。继续贯彻落实积极的就业政策，进一步扩大就业，促进再就业，新增城镇就业4.5万人，下岗失业人员再就业1.54万人。落实小额贷款政策，把好“贷、用、还”三个环节，重点帮扶下岗失业人员创业。继续加大对困难行业，困难群体再就业援助力度，对“4050”下岗失业人员开展专项服务，安排好他们的基本生活。认真做好失地农民就业和社会保障工作，建立失地农民失业登记制度，强化对失地农民再就业服务。统筹城乡就业，建立城乡协调发展的就业工作机制，积极探索建立统一开放、竞争有序的城乡劳动力市场，以解决农村劳动力进城就业“打工难、维权难、发展难”为重点，改善农村富余劳动力转移的外部环境。重视和加强就业技能培训工作，实施工业园区技术岗位对接、高技能人才培养样板、岗位成才示范和小企业孵化工程四大工程，提高劳动者就业能力和职业转换能力，扶持创办创业培训孵化基地、职业介绍和再就业培训。加强扶贫开发工作，实施好移民扶贫工程，确保完成移民任务。加强环境保护和生态建设，严把建设项目环保审批关，加大环保监督和执法力度，以水污染防治为重点，加强工业和城市污染治理，加强农村面源污染治理，加强饮用水源地保护。注重能源资源节约和合理利用，大力发展循环经济。

努力保持社会稳定。深入推进“和谐平安上饶”建设，高度重视处理好农村征地、城镇房屋拆迁、国有企业改制、案件审理等涉及群众切身利益的问题。健全正确处理人民内部矛盾的工作机制，建立和完善群体性事件的预警机制、化解矛盾的排查调处机制和运转高效的处置工作机制。做好人民群众来信来访工作，利用政策、法律、经济、行政等手段，采取教育、协商、调解等方法，依法及时合理地处理群众反映的问题。继续推进社会治安综合治理，深化全民普法教育，依法严厉打击危害公共利益和国家安全的各项犯罪行为。全面落实安全生产责任制，推行安全生产风险抵押金制度，毫不放松高危行业与领域的安全监督，加强学校、幼儿园、娱乐场所等人群密集区域的安全监督工作，防止事故发生，确保人民群众生命财产安全。

三、提高政府行政能力，建设人民满意的政府

按照“为民、务实、清廉”要求，以提高行政能力为重点，进一步加强政府自身建设，使各级政府工作为人民服务、对人民负责、受人民监督、让人民满意。

加快政府职能转变，推进管理创新。全面履行政府职能，在继续搞好经济调节、加强市场监管的同时，更加注重履行社会管理和公共服务职能，继续推行政企分开、政资分开、政事分开，进一步理顺政府与企业、政府与市场、政府与社会的关系；在加强和改进政府管理的同时，更加注重建设服务型政府。认真贯彻实施行政许可法，深化行政审批制度改革，继续做好行政审批事项的清理、规范和衔接工作，加强对行政审批行为的监管和后续监管。创新政府管理方式，增强按国际惯例办事和运用市场手段解决问题的能力，进一步推行政务公开，加快电子政务建设，扩大网上办公、信息公布和文件查阅的范围，增强政府工作透明度，提高政府公信力和办事效率。

坚持科学民主决策，推进依法行政。各级政府要围绕建设法治政府的目标，认真贯彻国务院《全面推进依法行政实施纲要》，带头维护宪法和法律的权威，严格按照法定权限和程序行使权力、履行职能，在行政工作中依法保障人民群众的合法权益。加快建立权责明确、行为规范、监督有效、保障有力的行政执法体制，保证严格执法、公正执法和文明执法，切实解决有法不依、执法不公、多头执法以及某些执法人员自由裁量权过大等群众反映强烈的问题。完善行政监督机制，强化对行政行为的监督，推行行政执法评议考核制和过错责任追究制，做到有权必有责、用权受监督、违法受追究、侵权须赔偿。坚持依法决策、科学决策、民主决策，健全社会公示、社会听证制度，完善公众参与、专家论证和政府决策相结合的行政决策机制，让广大人民群众广泛参与到公共管理事务上来。认真实施村民自治法，广泛推行村务公开，搞好村委会换届工作。自觉接受人民代表大会及其常委会的工作监督和法律监督；自觉接受人民政协的民主监督，认真办理人大代表议案、建议和政协委员提案。积极加强与各民主党派、工商联、无党派人士和各人民团体的联系，支持工会、共青团、妇联等群众性组织开展工作。全面贯彻党的民族、宗教政策，切实做好侨务和对台工作。

弘扬求真务实精神，推进政风建设。各级政府要牢固树立以人为本、执政为民的思想，把实现好、维护好、发展好人民群众的根本利益作为一切工作的出发点和落脚点。牢固树立科学发展观和正确政绩观，大力弘扬求真务实精神，坚决反对弄虚作假行为，绝不允许造假数字，搞所谓的“政绩工程”。认真开展保持共产党员先进性教育活动，把教育成果落实到推进政府各项工作上来。继续开展政务环境评议评价活动，推动政府各部门改进工作，提高服务质量和办事效率。时刻牢记“两个务必”，坚决反对骄傲自满、奢侈浪费。完善政绩考核办法，探索建立政府绩效评估体系和经济社会发展综合评价体系。加强与人民群众的密切联系，大兴调查研究之风，及时发现和处理实际工作中的新情况、新问题。加强制度约束，建设一支高素质的公务员队伍。进一步加强反腐倡廉工作，政府全体工作人员要常修为政之德，常思贪欲之害，常怀律己之心，勤政廉政，奉公守法，甘当人民的好公仆。

各位代表，新的一年充满希望和挑战，新的任务更加艰巨而繁重。我们深感肩负的责任重大而光荣，人民群众对政府的信任和支持，给了我们无穷的力量和必胜的信心。让我们在省委、省政府和市委的领导下，高举邓小平理论和“三个代表”重要思想伟大旗帜，解放思想，开拓创新，求真务实，扎实苦干，为全面完成今年各项任务、推进赣浙闽皖四省交界区域中心城市和快速发展地区建设而努力奋斗！
